# Macrovascular damage in polymyalgia rheumatica: integrated vascular phenotyping in the POLYMYACARD cohort

**DOI:** 10.1186/s13075-026-03886-8

**Published:** 2026-09-15

**Authors:** Sarah Wagner, Anna-Lena Zang, Markus Scheppers, Clio Mavragani, Jisna Paul, Ioannis Parodis, Dionysis Nikolopoulos, Andreas Schwarting, Konstantinos Triantafyllias

**Affiliations:** 1 Department of Internal Medicine I, Division of Rheumatology and Clinical Immunology, Johannes Gutenberg University Medical Center, Langenbeckstr. 1, Mainz, 55131 Germany; 2 Institute of Medical Biostatistics, Epidemiology and Informatics (IMBEI), University Medical Center, Johannes Gutenberg University, Mainz, Germany; 3 Department of Physiology, School of Medicine, National and Kapodistrian University of Athens, Athens, 11527 Greece; 4 Rheumatology and Immunology, Ohio State University Wexner Medical Center, Columbus, OH USA; 5 Division of Rheumatology, Department of Medicine Solna, Karolinska Institutet, Center for Molecular Medicine (CMM), Karolinska University Hospital, Stockholm, Sweden; 6 Department of Rheumatology, Faculty of Medicine and Health, Örebro University, Örebro, Sweden

**Keywords:** Polymyalgia Rheumatica, Aortic Stiffness, Carotid Doppler Ultrasound, Pulse Wave Velocity, Macroangiopathy, Cardiovascular Risk

## Abstract

**Background:**

To evaluate, for the first time, a wide combination of oscillometric and sonographic markers of macrovascular damage in patients with polymyalgia rheumatica (PMR), using carotid-femoral pulse wave velocity (cfPWV) and carotid greyscale/colour Doppler ultrasound (GSUS/CDUS). Additionally, to explore associations between these markers, traditional cardiovascular (CV) risk factors, and inflammatory parameters.

**Methods:**

Aortic stiffness was assessed by cfPWV. GSUS measured carotid intima-media thickness (cIMT), plaque presence, and cumulative plaque area. CDUS assessed haemodynamic/compliance surrogates, including resistance index (RI), pulsatility index (PI) in the common (CCA), internal (ICA), and external (ECA) carotid arteries. Multivariable linear regression (MLR) and propensity score matching (PSM) adjusted for confounding.

**Results:**

cfPWV was obtained in 72 PMR patients; 26 also underwent GSUS/CDUS. Compared with controls, PMR patients had higher cfPWV (p_adj_=0.012), increased RI (p_adj_=0.020), and PI (p_adj_=0.027) in the ECA after MLR and PSM. PSM further indicated a higher prevalence of subclinical arteriosclerosis in PMR [13/16 (81.3%) vs. 7/16 (43.8%); adjusted *p* = 0.028]. Plaque area was greater in patients with elevated C-reactive protein [0.41 (0.13–1.07) vs. 0.05 (0.00–0.31); *p* = 0.016]. cfPWV and carotid markers correlated with age (cfPWV *r* = 0.588, *p* < 0.001; cIMT *r* = 0.542, *p* = 0.004; plaques *r* = 0.662, *p* < 0.001) and mean arterial pressure (cfPWV ρ = 0.247, *p* = 0.038; cIMT *r* = 0.515, *p* = 0.020; plaques *r* = 0.596, *p* = 0.006).

**Conclusions:**

Patients with PMR exhibited increased aortic stiffness and elevated carotid pulsatility and resistance in the ECA compared with controls, independent of traditional CV risk factors. These non-invasive vascular markers may help identify PMR patients at increased CV risk and support integrated CV risk assessment in clinical care.

**Supplementary Information:**

The online version contains supplementary material available at https://doi.org/10.1186/s13075-026-03886-8.

## Introduction

Polymyalgia rheumatica (PMR) is an inflammatory autoimmune disease that predominantly affects older individuals, occurring two to three times more frequently in women than in men [1]. Its clinical relevance lies in its profound impact on quality of life due to symptoms such as symmetric pain in the shoulder and pelvic girdles, prolonged morning stiffness, and systemic manifestations like fever, weight loss, and depression [1, 2]. Moreover, PMR is frequently associated with giant cell arteritis (GCA), with both conditions sharing substantial clinical and pathophysiological overlap [2].

The diagnosis of PMR is typically based on clinical symptoms and supported by inflammatory markers, but it remains challenging due to the absence of specific biomarkers, heterogeneous symptom presentation, and overlap with other diseases [1, 3]. The 2012 European League Against Rheumatism (EULAR) and American College of Rheumatology (ACR) classification criteria, mainly developed for research purposes, facilitate disease classification [4]. Imaging, especially musculoskeletal ultrasonography, further improves diagnostic consistency [1].

PMR extends beyond its musculoskeletal manifestations, with growing evidence pointing to an increased risk of cardiovascular disease (CVD) [5, 6]. Persistent systemic inflammation is a recognized driver of atherosclerosis and may contribute to increased cardiovascular (CV) risk in autoimmune diseases [7].

This augmented CV risk in PMR has been demonstrated in several studies [5, 6, 8]. A large-scale study from the UK, analysing 3,249 PMR patients and 12,735 matched controls, reported an increased incidence of major CVD events of 12.2 per 1,000 person-years, with an adjusted hazard ratio (HR) of 2.6 (95% CI: 2.4–2.9) [9]. Similarly, a cohort study with 353 PMR patients demonstrated an elevated risk for peripheral artery disease compared to the general population [8]. Furthermore, a systematic review and meta-analysis by Ungprasert et al. [5], encompassing five observational retrospective studies, found an increased risk of coronary artery disease in PMR by a pooled risk ratio of 1.13.

However, not all studies revealed a higher CVD risk in PMR patients, and overall mortality has been shown to not differ from the general population [6, 10]. For instance, a systematic review by Hancock et al. [6] found increased CV risk in retrospective studies, but not in prospective ones, attributing the heterogeneous findings to inconsistent exclusion of GCA across studies, as GCA is independently associated with increased vascular risk. Furthermore, variability in study design, as well as heterogeneity in endpoint definitions and outcome ascertainment, further limit comparability between studies. These discrepancies emphasize not only the necessity for further evaluation but also the importance of early CV risk assessment in PMR patients. Established CVD markers in the general population, for example, the Systematic Coronary Risk Evaluation (SCORE), the Framingham Risk Score, or the Prospective Cardiovascular Münster (PROCAM) risk Score, have been shown to underestimate the CVD risk in autoimmune rheumatic diseases (ARDs), as they do not account for disease-specific factors such as systemic inflammation, chronic glucocorticoid therapy, and associated comorbidities [11, 12]. Consequently, the evaluation of alternative surrogate markers for CV risk is warranted to refine CVD screening in PMR [13]. In recent years, our research group and others have evaluated a panel of novel CV markers in patients with various arthritides, connective tissue diseases, and vasculitides [14–21]. CfPWV, known as the gold standard for measuring aortic stiffness, has gained prominence as a valuable tool for CVD risk assessment as it has a strong predictive value for future CV events and all-cause mortality [22–24]. Carotid intima-media thickness (cIMT) and carotid plaque assessment are additional markers for CVD risk evaluation, enabling the detection of subclinical atherosclerosis and showing strong associations with CV events such as myocardial infarction and stroke [25]. Complementary functional information is provided by the Doppler parameters, resistance index (RI), and pulsatility index (PI) of the carotid arteries. RI and PI have been associated with white matter hyperintensity in MRI imaging, cognitive impairment in older people and stroke patients, potentially reflecting small vascular disease [26, 27].

Data regarding the evaluation of novel CVD markers in PMR, including aortic stiffness, carotid compliance, and subclinical carotid atherosclerosis (SCA), are scarce. Therefore, this study aimed to comprehensively assess a broad panel of novel CV surrogate markers in PMR for the first time. Furthermore, we explored their associations with traditional CV risk factors and inflammatory disease characteristics.

## Methods

### Study population

This cross-sectional observational study included 72 consecutive patients with a clinical diagnosis of PMR based on the 2012 EULAR/ACR provisional classification criteria. Patients were recruited in the context of the POLYMYACARD (POLYMYalgia Associated CARDiovascular risk) study, a part of the multicentre German CARD cohorts. Exclusion criteria were age < 50 or > 85 years, inability to provide informed consent, pregnancy, known aortic or carotid artery stenosis, end-stage renal disease (estimated glomerular filtration rate (eGFR) < 30 ml/min/1.73 m²), body mass index (BMI) > 45 kg/m², and the presence of GCA based on available clinical data. All participants provided written informed consent. The study was approved by the Rhineland-Palatinate medical board and conducted in accordance with the Declaration of Helsinki (approval number:13762_2).

### Clinical and laboratory assessment

Patient data were collected at enrolment, including age, sex, body mass index (BMI), medical history (hypertension, diabetes mellitus, smoking), medication use (glucocorticoids, statins, antihypertensives, disease-modifying antirheumatic drugs (DMARDs), non-steroidal anti-inflammatory drugs (NSAIDs), and direct oral anticoagulants (DOACs). Laboratory parameters included C-reactive protein (CRP), erythrocyte sedimentation rate (ESR), lipid profile, and estimated glomerular filtration rate (eGFR).

### Pulse Wave Velocity (PWV) measurement and sonography

Aortic stiffness was assessed non-invasively via carotid-femoral pulse wave velocity (cfPWV) using the Vicorder^®^ system (SMT Medical, Würzburg, Germany). Examinations were conducted by trained staff blinded to patient clinical characteristics. Measurements were performed according to expert consensus protocols and manufacturer instructions. The pulse wave travel distance was measured from the right common carotid artery (CCA) to the femoral artery, with pulse transit time determined using pressure cuffs. The cfPWV was calculated by dividing the distance by transit time, applying a correction factor of 0.8 [28]. The average of three consecutive measurements was used for analysis. A cfPWV value > 10 m/s was considered indicative of increased CVD risk [28].

Grey-scale ultrasound (GSUS) and colour Doppler ultrasound (CDUS) examinations were performed in a subgroup by an experienced examiner (K.T.), certified trainer of the German Society for Ultrasound in Medicine (DEGUM). The examinations were conducted using a linear transducer (4–15 MHz) on a MyLab9-US device (Esaote).

Carotid intima–media thickness was measured manually on longitudinal B-mode images of the common carotid artery, 1 cm proximal to the carotid bulb, at end-diastole. Measurements were obtained on the far wall, from the leading edge of the lumen–intima interface to the leading edge of the media–adventitia interface, with the calipers positioned perpendicular to the arterial wall. Three consecutive measurements were performed on each side, and the highest value was included in the statistical analysis. Plaques defined as localized thickening > 1.2 mm were bilaterally examined in the CCA and carotid bulb [29]. SCA was defined as cIMT > 0.9 mm and/or the presence of one or more plaques [29]. In the same subgroup, Doppler ultrasonography of the external carotid artery (ECA), ICA, and CCA was conducted using transverse and longitudinal planes, examining peak systolic velocity (PSV) and end-diastolic velocity (EDV). The device automatically calculated the RI and PI according to Pourcelot’s [$\mathrm{RI}=\left(\mathrm{PSV}-\mathrm{EDV}\right)/\mathrm{PSV}$] and Gosling’s formula [$\left(\mathrm{PSV}-\mathrm{EDV}\right)/\text{mean flow velocity}\left(\mathrm{MFV}\right)$].

### Statistical analysis

The assumption of normality was determined by the Shapiro-Wilk test. Normally distributed continuous variables were presented as mean (± standard deviation) and non-normally distributed continuous variables as median (interquartile range). For unmatched data, independent-samples t-tests were used to evaluate the differences across groups for normally distributed variables, and Mann-Whitney U tests for skewed continuous variables. In the case of three groups, one-way ANOVA for normally distributed continuous variables or the Kruskal-Wallis test for skewed continuous variables were used to compare differences across the groups. Categorical variables, demonstrated in relative (n) and absolute (%) numbers, were analysed for significant differences by Chi^2^ test. In all performed tests, a *p*-value < 0.05 was considered statistically significant.

Spearman’s rho assessed the direction and strength of correlations in non-normally distributed variables, whereas Pearson’s r was used for normally distributed variables. To adjust for confounders, linear regression and propensity score matching (PSM) were performed. A priori, we chose to adjust for age, gender, diabetes, arterial hypertension, and nicotine use due to their known confounding effects and their unequal distribution between groups. IBM SPSS^®^ 29.0.2.0 software was used for statistical analyses. PSM and subsequent paired cohort analyses were performed using R version 4.5.2. Propensity scores were estimated via logistic regression, and genetic matching with replacement (GenMatch function) was applied to the total cohort to maximise statistical power and optimise covariate balance. Propensity scores were estimated via logistic regression, and genetic matching with replacement (GenMatch function) was applied to the total cohort to maximise statistical power and optimise covariate balance. In the sonography subgroup, genetic matching without replacement was used to avoid repeated use of controls in the smaller sample size. Matched datasets were exported for subsequent analyses in SPSS.

## Results

CfPWV was assessed in 72 patients and 143 controls. In addition, CDUS and GSUS examinations were performed in a subgroup of 26 patients and 88 controls. Descriptive characteristics of patients and controls are summarized in Tables 1 and 2. Tables 3, 4, 5, 6 and 7 demonstrate the correlations between CVD surrogates and patient and disease characteristics.

**Table 1:** Descriptive characteristics by full group

All subjects	Patients (*n* = 72)	Controls (*n* = 143)	*p* value
Age^a^ *(years)*	69.00 (60.00–74.00)	53.00 (43.00–59.00)	< 0.001***
Gender *(female)*	44 (61.1%)	122 (85.3%)	< 0.001***
BMI^a^ *(kg/m*^*2*^*)*	26.40 (23.00–31.00)	25.40 (22.04–28.28)	0.103
Height^a^ *(cm)*	167.00 (160.00-174.00)	168.00 (163.00-172.25)	0.581
Weight^a^ *(kg)*	76.50 (62.25-88.00)	71.50 (64.00–80.00)	0.118
Arterial hypertension *(yes)*	46 (63.9%)	37 (27.6%)	< 0.001***
Diabetes mellitus *(yes)*	19 (26.8%)	3 (2.2%)	< 0.001***
Nicotine use *(smoker)* *(ex-smoker)*	8 (11.1%) 9 (12.5%)	26 (19.4%) 8 (6.00%)	0.111
HDL^a^ *(mg/dl)*	67.00 (49.00–82.00)	63.00 (54.75-76.00)	0.700
LDL^b^ *(mg/dl)*	120.45 (38.60)	128.29 (35.34)	0.198
Total cholesterol^b^ *(mg/dl)*	203.10 (49.35)	209.57 (41.57)	0.329
TAG^a^ *(mg/dl)*	102.00 (77.50-142.50)		
SAP-DAP^a^ *(mmHg)*	50.00 (40.00–60.00)	45.00 (40.00–50.00)	0.002*
SAP^a^ *(mmHg)*	130.00 (120.00-145.00)	125.00 (115.00-132.00)	0.003*
DAP^a^ *(mmHg)*	80.00 (70.00–85.00)	80.00 (70.00–86.00)	0.794
MAP^a^ *(mmHg)*	96.67 (90.00-103.33)	95.00 (86.67-102.67)	0.078
cfPWV^b^ *(m/s)*	9.21 (1.81)	7.19 (1.38)	< 0.001***
Prednisolone equivalent *(mg)*	6.50 (0.25-15.00)		
DMARDs *(yes)*	15 (20.8%)		
Statin therapy *(yes)*	20 (27.8%)	5 (3.7%)	< 0.001***
eGFR^b^ *(ml/min)*	76.96 (19.24)	90.87 (16.13)	< 0.001***
ESR^a^ *(mm/h)*	30.00 (12.50–59.00)	12.00 (8.00–18.00)	< 0.001***
CRP positive *(yes)*	42 (59.2%)	0 (0%)	< 0.001***
Rheumatic factor *(yes)*	17 (24.6%)		
Disease duration^a^ *(months)*	6.50 (2.25–22.75)		
Morning stiffness *(yes)*	35 (66.0%)		
Neck stiffness *(yes)*	15 (71.4%)		
Chewing pain *(yes)*	1 (3.7%)		
Bilateral shoulder pain *(yes)*	65 (98.5%)		
Myalgia *(yes)*	71 (100%)		
Arthralgia *(yes)*	39 (83.0%)		
Arthritis *(yes)*	10 (27.8%)		
Pelvic gridle pain *(yes)*	62 (93.9%)		
B-symptomatic *(yes)*	7 (9.7%)	0 (0.0%)	< 0.001***
Cardiovascular events *(yes)*	20 (27.8%)		

*BMI* Body Mass Index, *HDL* high-density lipoprotein, *LDL* low-density lipoprotein, *TAG* triacylglycerol, *SAP* systolic arterial pressure, *DAP* diastolic arterial pressure, *MAP* mean arterial pressure, *cfPWV* carotid-femoral pulse wave velocity, *DMARDs* disease-modifying-anti-rheumatic drugs, *eGFR* estimated glomerular filtration rate, *ESR* erythrocyte sedimentation rate, *CRP* C-reactive protein

*-*** Significant between the two groups (*p <0.05; ***p <0.001)

^a^Non-normal distribution: presentation as median (interquartile range)

^b^Normal distribution: presentation as mean (S.D.)

**Table 2:** Descriptive characteristics by subgroup

All subjects	Patients (*n* = 26)	Controls (*n* = 88)	*p* value
Age^a^ *(years)*	70.50 (60.00-74.75)	55.00 (48.00-6100)	< 0.001***
Female *(yes)*	13 (50%)	71 (80.7%)	0.002*
BMI^a^ *(kg/m*^*2*^*)*	28.70 (23.00-32.30)	26.32 (23.53–29.96)	0.798
Height^a^ *(cm)*	170.50 (164.75-176.75)	166.00 (162.00-172.00)	0.039*
Weight^a^ *(kg)*	84.00 (68.75–90.25)	74.50 (65.00–85.00)	0.093
Arterial hypertension *(yes)*	17 (68.0%)	30 (36.1%)	0.006*
Diabetes mellitus *(yes)*	9 (36.0%)	3 (3.6%)	< 0.001***
Nicotine use *(smoker)* *(ex-smoker)*	5 (20%) 4 (16%)	17 (20.5%) 5 (20%)	0.671
HDL^a^ *(mg/dl)*	51.00 (47.00–75.00)	61.00 (53.00-75.50)	0.113
LDL^a^ (*mg/dl)*	123.00 (114.50-135.50)	135.00 (104.50–157.00)	0.218
Total cholesterol^b^ *(mg/dl)*	200.56 (50.06)	210.45 (39.03)	0.318
TAG^a^ *(mg/dl)*	124.00 (83.00-200.50)		
SAP-DAP^a^ *(mmHg)*	52.50 (40.00-63.75)	45.00 (40.00–50.00)	0.055
SAP^b^ *(mmHg)*	132.75 (16.97)	126.71 (13.73)	0.121
DAP^a^ *(mmHg)*	80.00 (76.35–88.75)	80.00 (75.00–90.00)	0.901
MAP^a^ *(mmHg)*	99.00 (91.25–104.50)	99.00 (90.00-103.08)	0.447
cIMT^a^ *(mm)*	0.99 (0.82–1.20)	0.78 (0.67–0.91)	< 0.001***
Plaques *(yes)*	21 (80.8%)	28 (31.8%)	< 0.001***
Plaque count^a^ *(median)*	2.00 (1.00–3.00)	0.00 (0.00–1.00)	< 0.001***
Plaque count	0*5 (19.2%) 1* 7 (26.9%) 2* 4 (15.4%) 3*4 (15.4%) 4*2 (7.7%) 5*1 (3.8%) 6*2 (7.7%) 7*0(0.0%) 8*0 (0.0%) 9*1 (3.8%)	0*60 (68.2%) 1* 16 (18.2%) 2*8 (9.1%) 3*1 (1.1%) 4*1 (1.1%) 5*0 (0.0%) 6*0 (0.0%) 7*1 (1.1%) 8*1 (1.1%) 9*0(0.0%)	< 0.001***
Calcification area^a^ *(cm*^*2*^*)*	0.12 (0.02–0.41)	0.00 (0.00-0.06)	< 0.001***
SCA *(yes)*	22 (84.6%)	36 (44.8%)	< 0.001***
CCA-PI^a^	1.80 (1.61–1.95)	1.61 (1.39–1.84)	0.056
CCA-RI^a^	0.77 (0.74–0.82)	0.74 (0.69–0.77)	0.008*
ICA-PI^a^	1.50 (1.21–1.88)	1.22 (0.96–1.54)	0.004*
ICA-RI^b^	0.74 (0.11)	0.66 (0.09)	< 0.001***
ECA-PI^a^	2.90 (2.41–3.20)	1.99 (1.69–2.39)	< 0.001***
ECA-RI^a^	0.89 (0.85–0.92)	0.80 (0.75–0.84)	< 0.001***
Prednisolone equivalent^a^ *(mg)*	7.50 (1.00-12.50)		
DMARDs *(yes)*	6 (24.0%)		
Statin therapy *(yes)*	7 (28.0%)	5 (6.0%)	0.002*
eGFR^a^ *(ml/min)*	72.73 (57.96–83.34)	84.03 (75.57–96.97)	< 0.001***
ESR^a^ *(mm/h)*	20.00 (10.00–34.00)	12.00 (8.00–18.00)	0.027*
CRP positive *(yes)*	13 (54.2%)	0 (0%)	< 0.001***
Rheumatic factor *(yes)*	6 (27.3%)		
Disease duration^a^ *(months)*	8.50 (4.50–25.00)		
Morning stiffness *(yes)*	13 (65.0%)		
Neck stiffness *(yes)*	7 (58.3%)		
Chewing pain *(yes)*	0 (0%)		
Bilateral shoulder pain *(yes)*	24 (100%)		
Myalgia *(yes)*	26 (100%)		
Arthralgia *(yes)*	17 (85.0%)		
Arthritis *(yes)*	2 (16.7%)		
Pelvic gridle pain *(yes)*	19 (90.5%)		
Malignancy *(yes)*	2 (7.7%)		
B-symptomatic *(yes)*	5 (19.2%)		
Cardiovascular events *(yes)*	6 (26.1%)		
Other autoimmune disease *(yes)*	2 (9.1%)		

*BMI* Body Mass Index, *HDL* high-density lipoprotein, *LDL* low-density lipoprotein, *TAG* triacylglycerol, *SAP *systolic arterial pressure, *DAP* diastolic arterial pressure, *MAP* mean arterial pressure, *cIMT* carotid intima-media thickness, *SCA *subclinical carotid atherosclerosis, *CCA *common carotid artery, *ICA* internal carotid artery, *ECA* external carotid artery, *RI* resistance index, *PI* pulsatility index, *DMARDs *disease-modifyinganti-rheumatic drugs, *eGFR* estimated glomerular filtration rate, *ESR* erythrocyte sedimentation rate, *CRP* C-reactive protein

*-*** Significant between the two groups (*p <0.05; ***p <0.001)

^a^Non-normal distribution: presentation as median (interquartile range)

^b^Normal distribution: presentation as mean (S.D.)

**Table 3:** Associations between cfPWV values and patients’ characteristics

	cfPWV				
	Rho/*r*	*p*-value		Mean(S.D.)/median (IQR)	*p*-value
Age^a^ *(years)*	0.588	< 0.001***	Gender^a^		
BMI^a^ *(kg/m*^*2*^*)*	-0.122	0.305	Male	9.44 (7.05–10.57)	0.492
			Female	9.23 (8.20–10.70)	
Height^a^ *(cm)*	-0.215	0.072	Arterial hypertension^a^		
Weight^a^ *(kg)*	-0.224	0.058			
			No	9.20 (6.88–10.85)	0.647
			Yes	9.30 (8.15–10.64)	
HDL^a^ *(mg/dl)*	0.216	0.120	Diabetes mellitus^a^		
LDL^a^ *(mg/dl)*	-0.093	0.507	No	9.11 (1.95)	0.443
			Yes	9.49 (1.46)	
Total cholesterol^a^ *(mg/dl)*	-0.023	0.847	Nicotine use^b^		
			No	9.28 (1.80)	0.854
TAG^a^ *(mg/dl)*	-0.112	0.359	Smoker	8.88 (2.12)	
			Ex-nicotine use	9.17 (1.81)	
SAP-DAP^a^ *(mmHg)*	0.351	0.003*			
SAP^a^ *(mmHg)*	0.331	0.005*	DMARD^a^		
DAP^a^ *(mmHg)*	0.053	0.660	No	9.20 (8.13–10.51)	0.589
			Yes	9.36 (6.51–10.80)	
MAP^a^ *(mmHg)*	0.247	0.038*	Statin therapy^b^		
			No	9.27 (1.82)	0.675
Disease duration *(months)*	0.052	0.667	Yes	9.07 (1.84)	
Prednisolone equivalent^a^ *(mg)*	0.038	0.754	DOAC^b^		
			No	9.20 (1.81)	0.824
eGFR^a^ *(ml/min)*	0.102	0.405	Yes	9.41 (2.20)	
ESR^a^ *(mm/h)*	0.148	0.224	Cardiovascular events^a^		
			No	9.20 (7.99–10.32)	0.188
			Yes	9.52 (8.09–11.22)	

Quantitative characteristics: Spearman’s (a) (non-normal distribution, rho) and Pearson’s (b) (normal distribution, r). Qualitative characteristics: (a) non-normal distribution: presentation as median (interquartile range); (b) normal distribution: presentation as mean (standard deviation)

*cfPWV* carotid-femoral pulse wave velocity, *BMI* Body Mass Index, *HDL* high-density lipoprotein, *LDL* low-density lipoprotein, *TAG* triacylglycerol, *SAP*systolic arterial pressure, *DAP* diastolic arterial pressure, *MAP* mean arterial pressure, *DMARDs *disease-modifyinganti-rheumatic drugs, *eGFR* estimated glomerular filtration rate,  *ESR* erythrocyte sedimentation rate, *CRP* C-reactive protein

*-*** Significant between the two groups (*p <0.05; ***p <0.001)

**Table 4:** Associations between Calcification area/cIMT and patients’ characteristics

	Calcification area			cIMT	
	Rho/*r*	*p*-value		Rho/*r*	*p*-value
Age^a^ *(years)*	0.662	< 0.001***	Age^a^ *(years)*	0.542	0.004*
BMI^a^ (*kg/m*^*2*^)	0.232	0.286	BMI^a^ *(kg/m*^*2*^*)*	0.353	0.083
Height^a^ *(cm)*	-0.373	0.096	Height^b^ *(cm)*	-0.069	0.760
Weight^a^ *(kg)*	0.056	0.811	Weight^b^ *(kg)*	0.432	0.044*
HDL^a^ (*mg/dl)*	0.368	0.161	HDL^a^ *(mg/dl)*	0.170	0.514
LDL^a^ (*mg/dl)*	0.372	0.156	LDL^a^ *(mg/dl)*	-0.194	0.455
Total cholesterol^a^ *(mg/dl)*	-0.035	0.875	Total cholesterol^b^ *(mg/dl)*	-0.334	0.103
TAG^a^ *(mg/dl)*	0.106	0.629	TAG^a^*(mg/dl)*	0.260	0.210
SAP-DAP ^a^ *(mmHg)*	0.687	< 0.001***	SAP-DAP^b^*(mmHg)*	0.321	0.168
SAP ^a^ *(mmHg)*	0.686	< 0.001***	SAP^b^ *(mmHg)*	0.422	0.064
DAP^a^ *(mmHg)*	0.229	0.331	DAP^a^ *(mmHg)*	0.305	0.191
MAP^a^ (mmHg)	0.596	0.006*	MAP^a^ *(mmHg)*	0.515	0.020*
cfPWV^a^ *(m/s)*	0.812	< 0.001***	cfPWV^a^ *(m/s)*	0.519	0.019*
cIMT ^a^ *(mm)*	0.582	0.003*	cIMT^b^ *(mm)*		
Calcification area^a^			Calcification area^a^	0.582	0.003*
CCA-PI ^a^	0.040	0.864	CCA-PI^b^	-0.125	0.588
CCA-RI ^a^	0.158	0.493	CCA-RI^b^	-0.130	0.575
ICA-PI^a^	-0.019	0.935	ICA-PI^a^	-0.187	0.416
ICA-RI ^a^	0.052	0.823	ICA-RI^b^	-0.204	
ECA-PI^a^	0.008	0.971	ECA-PI^a^	-0.075	0.745
ECA-RI ^a^	0.071	0.767	ECA-RI^b^	-0.122	0.607
Prednisolone equivalent^a^ *(mg)*	-0.167	0.447	Prednisolone equivalent^a^ *(mg)*	0.196	0.347
eGFR ^a^ *(ml/min)*	-0.229	0.294	eGFR^b^ (ml/min)	-0.154	0.463
ESR^a^ *(mm/h)*	0.017	0.940	ESR^a^ (mm/h)	0.191	0.382
Disease duration^a^ *(months)*	-0.037	0.864	Disease duration^a^ *(months)*	-0.106	0.606

	Mean (S.D.)/ Median (IQR)	*p*-value		Mean (S.D.)/ Median (IQR)	*p*-value
Gender ^a^			Gender^b^		
*Male* *Female*	0.24 (0.00-0.41) 0.09 (0.05–0.42)	0.908	*Male* *Female*	1.06 (0.30) 0.96 (0.25)	0.343
Arterial hypertension ^a^			Arterial hypertension^b^		
*No* *Yes*	0.00 (0.00-0.05) 0.38 (0.09–0.59)	0.002*	*No* *Yes*	0.84 (0.28) 1.08 (0.25)	0.053
Diabetes mellitus ^a^			Diabetes mellitus^b^		
*No* *Yes*	0.09 (0.00-0.41) 0.38 (0.13–0.42)	0.098	*No* *Yes*	0.94 (0.26) 1.12 (0.29)	0.109
Nicotine use ^a^			Nicotine use^a^		
*No* *Yes* *Ex-nicotine use*	0.09 (0.00-0.41) 0.09 (0.04–0.14) 0.74 (0.37–1.36)	0.064	*No* *Yes* *Ex-nicotine use*	0.98 (0.76–1.08) 0.93 (0.82–0.98) 1.25 (1.20–1.65)	0.042*
DMARDs ^a^			DMARDs^a^		
*No* *Yes*	0.31 (0.06–0.53) 0.03 (0.00-0.23)	0.149	*No* *Yes*	0.99 (0.93–1.20) 0.77 (0.54–0.96)	0.024*
Statin therapy ^a^			Statin therapy^b^		
*No* *Yes*	0.13 (0.00-0.41) 0.25 (0.04–0.68)	0.587	*No* *Yes*	0.91 (0.22) 1.25 (0.27)	0.003*
CRP positive ^a^			CRP positive^a^		
*No* *Yes*	0.05 (0.00-0.31) 0.41 (0.13–1.07)	0.016*	*No* *Yes*	0.88 (0.72–1.20) 0.99 (0.90–1.25)	0.369
Cardiovascular events ^a^			Cardiovascular events^b^		
*No* *Yes*	0.13 (0.05–0.41) 0.39 (0.07–1.17)	0.275	*No* *Yes*	0.93 (0.21) 1.22 (0.34)	0.020*

Quantitative characteristics: Spearman’s (a) (non-normal distribution, rho) and Pearson’s (b) (normal distribution, r). Qualitative characteristics: (a) non-normal distribution: presentation as median (interquartile range); (b) normal distribution: presentation as mean (standard deviation)

*cfPWV* carotid-femoral pulse wave velocity, *BMI* Body Mass Index, *HDL* high-density lipoprotein, *LDL* low-density lipoprotein, *TAG* triacylglycerol, *SAP *systolic arterial pressure, *DAP* diastolic arterial pressure, *MAP* mean arterial pressure, *cIMT* carotid intima-media thickness, *CCA *common carotid artery, *ICA* internal carotid artery, *ECA* external carotid artery, *RI* resistance index, *PI* pulsatility index, *DMARDs *disease-modifying-anti-rheumatic drugs, *eGFR* estimated glomerular filtration rate, *ESR* erythrocyte sedimentation rate, *CRP* C-reactive protein

*-*** Significant between the two groups (*p <0.05; ***p <0.001)

**Table 5:** Associations between CCA-PI/ CCA-RI and patients’ characteristics

	CCA-PI			CCA-RI	
	Rho/*r*	*p*-value		Rho/*r*	*p*-value
Age^a^ *(years)*	0.371	0.098	Age^a^ *(years)*	0.593	0.005*
BMI^a^ *(kg/m*^*2*^*)*	-0.201	0.395	BMI^a^ *(kg/m*^*2*^*)*	-0.067	0.778
Height^b^ *(cm)*	-0.218	0.369	Height^b^ *(cm)*	-0.160	0.512
Weight^b^ *(kg)*	-0.417	0.076	Weight^b^ *(kg)*	-0.298	0.215
HDL^a^ *(mg/dl)*	0.089	0.761	HDL^a^ *(mg/dl)*	-0.049	0.869
LDL^a^ *(mg/dl)*	-0.106	0.719	LDL^a^ *(mg/dl)*	-0.013	0.964
Total cholesterol^b^ *(mg/dl)*	-0.078	0.744	Total cholesterol^b^ *(mg/dl)*	-0.207	0.380
TAG^a^ *(mg/dl)*	-0.187	0.429	TAG^a^ *(mg/dl)*	-0.192	0.417
SAP-DAP^b^ *(mmHg)*	-0.076	0.763	SAP-DAP^b^ *(mmHg)*	0.079	0.756
SAP^b^ *(mmHg)*	-0.103	0.685	SAP^b^ *(mmHg)*	0.102	0.686
DAP^a^ (*mmHg)*	-0.189	0.453	DAP^a^ (*mmHg)*	0.027	0.914
MAP^a^ *(mmHg)*	-0.084	0.739	MAP^a^ *(mmHg)*	0.211	0.400
cfPWV^a^ *(m/s)*	-0.176	0.486	cfPWV^a^ *(m/s)*	0.119	0.639
cIMT^b^ *(mm)*	-0.125	0.588	cIMT^b^ *(mm)*	-0.130	0.575
Calcification area^a^	0.040	0.864	Calcification area^a^	0.158	0.493
CCA-PI^b^			CCA-PI^b^	0.840	< 0.001***
CCA-RI^b^	0.840	< 0.001***	CCA-RI^b^		
ICA-PI^a^	0.607	0.004*	ICA-PI^a^	0.390	0.081
ICA-RI^b^	0.411	0.064	ICA-RI^b^	0.438	0.047*
ECA-PI^a^	0.327	0.148	ECA-PI^a^	0.339	0.133
ECA-RI^b^	0.418	0.067	ECA-RI^b^	0.557	0.011*
Prednisolone equivalent^a^ *(mg)*	-0.022	0.925	Prednisolone equivalent^a^ *(mg)*	0.006	0.978
eGFR^b^ *(ml/min)*	0.067	0.777	eGFR^b^ *(ml/min)*	-0.066	0.781
ESR^a^ (*mm/h)*	-0.071	0.781	ESR^a^ *(mm/h)*	-0.240	0.337
Disease duration^a^ *(months)*	-0.091	0.696	Disease duration^a^ (*months)*	-0.013	0.954

	Mean (S.D)/Median (IQR)	*p*-value		Mean (S.D)/Median (IQR)	*p*-value
Gender ^b^			Gender^b^		
*Male* *Female*	1.77 (0.34) 1.74 (0.33)	0.865	*Male* *Female*	0.78 (0.04) 0.76 (0.08)	0.463
Arterial hypertension ^b^			Arterial hypertension^a^		
*No* *Yes*	1.78 (0.40) 1.77 (0.30)	0.947	*No* *Yes*	0.74 (0.72–0.82) 0.78 (0.76–0.82)	0.336
Diabetes mellitus^b^			Diabetes mellitus^b^		
*No* *Yes*	1.74 (0.38) 1.82 (0.19)	0.652	*No* *Yes*	0.77 (0.06) 0.78 (0.05)	0.805
Nicotine use ^a^			Nicotine use^b^		
*No* *Yes* *Ex-nicotine use*	1.80 (1.73–1.93) 1.72 (1.30–1.99) 1.82 (1.71–1.87)	0.915	*No* *Yes* *Ex-nicotine use*	0.77 (0.07) 0.77 (0.04) 0.78 (0.01)	0.940
DMARDs ^b^			DMARDs^a^		
*No* *Yes*	1.81 (0.34) 1.67 (0.23)	0.418	No Yes	0.78 (0.75–0.82) 0.76 (0.73–0.82)	0.540
Statin therapy ^b^			Statin therapy^b^		
*No* *Yes*	1.74 (0.34) 1.88 (0.25)	0.464	No Yes	0.76 (0.06) 0.82 (0.03)	0.118
DOAC ^b^			DOAC^b^		
*No* *Yes*	1.71 (0.34) 1.96 (0.17)	0.131	*No* *Yes*	0.76 (0.06) 0.81 (0.02)	0.162
CRP positive ^a^			CRP positive^a^		
*No* *Yes*	1.74 (0.25) 1.80 (0.40)	0.348	*No* *Yes*	0.77 (0.73–0.82) 0.79 (0.76–0.82)	0.461
Cardiovascular events ^b^			Cardiovascular events^b^		
*No* *Yes*	1.68 (0.40) 1.92 (0.16)	0.096	*No* *Yes*	0.75 (0.07) 0.81 (0.03)	0.082

Quantitative characteristics: Spearman’s (a) (non-normal distribution, rho) and Pearson’s (b) (normal distribution, r). Qualitative characteristics: (a) non-normal distribution: presentation as median (interquartile range); (b) normal distribution: presentation as mean (standard deviation)

*cfPWV* carotid-femoral pulse wave velocity, *BMI* Body Mass Index, *HDL* high-density lipoprotein, *LDL* low-density lipoprotein, *TAG* triacylglycerol, *SAP *systolic arterial pressure, *DAP* diastolic arterial pressure, *MAP* mean arterial pressure, *cIMT* carotid intima-media thickness, *CCA *common carotid artery, *ICA* internal carotid artery, *ECA* external carotid artery, *RI* resistance index, *PI* pulsatility index, *DMARDs *disease-modifying-anti-rheumatic drugs, *eGFR* estimated glomerular filtration rate, *ESR* erythrocyte sedimentation rate, *CRP* C-reactive protein

*-*** Significant between the two groups (*p <0.05; ***p <0.001)

**Table 6:** Associations between ICA-PI/ CCA-RI and patients’ characteristics

	ICA-PI			ICA-RI	
	Rho/*r*	*p*-value		Rho/*r*	*p*-value
Age^a^ *(years)*	0.132	0.568	Age^a^ *(years)*	0.131	0.571
BMI^a^ *(kg/m*^*2*^*)*	-0.029	0.905	BMI^a^ *(kg/m*^*2*^*)*	0.045	0.852
Height^a^ *(cm)*	0.004	0.989	Height^b^ *(cm)*	0.077	0.755
Weight^a^ *(kg)*	-0.020	0.934	Weight^b^ *(kg)*	0.142	0.561
HDL^a^ *(mg/dl)*	0.066	0.822	HDL^a^ *(mg/dl)*	0.113	0.701
LDL^a^ (*mg/dl)*	-0.011	0.970	LDL^a^ (*mg/dl)*	0.126	0.668
Total cholesterol^a^ *(mg/dl)*	0.104	0.663	Total cholesterol^b^ *(mg/dl)*	0.309	0.185
TAG^a^ *(mg/dl)*	-0.232	0.326	TAG^a^ *(mg/dl)*	-0.169	0.477
SAP-DAP ^a^ *(mmHg)*	0.057	0.822	SAP-DAP^b^ *(mmHg)*	0.061	0.810
SAP ^a^ *(mmHg)*	-0.116	0.647	SAP^b^ *(mmHg)*	-0.159	0.529
DAP^a^ *(mmHg)*	-0.492	0.038*	DAP^a^ *(mmHg)*	-0.453	0.059
MAP^a^ *(mmHg)*	-0.359	0.144	MAP^a^ (mmHg)	-0.311	0.209
cfPWV^a^ *(m/s)*	-0.144	0.570	cfPWV^a^ *(m/s)*	-0.012	0.962
cIMT ^a^ (mm)	-0.187	0.416	cIMT^b^ *(mm)*	-0.204	0.375
Calcification area^a^	-0.019	0.935	Calcification area^a^	0.052	0.823
CCA-PI ^a^	0.607	0.004*	CCA-PI^b^	0.411	0.064
CCA-RI ^a^	0.390	0.081	CCA-RI^b^	0.438	0.047*
ICA-PI^a^			ICA-PI^a^	0.957	< 0.001***
ICA-RI ^a^	0.957	< 0.001***	ICA-RI^b^		
ECA-PI^a^	0.358	0.111	ECA-PI^a^	0.234	0.308
ECA-RI ^a^	0.577	0.008*	ECA-RI^b^	0.271	0.248
Prednisolone equivalent^a^ *(mg)*	0.213	0.368	Prednisolone equivalent^a^ *(mg)*	0.314	0.178
eGFR ^a^ *(ml/min)*	0.224	0.342	eGFR^b^ *(ml/min)*	0.001	0.997
ESR^a^ *(mm/h)*	-0.077	0.762	ESR^a^ *(mm/h)*	-0.094	0.712
Disease duration^a^ *(months)*	-0.078	0.738	Disease duration^a^ *(months)*	-0.092	0.692

	Median (IQR)/Mean (S.D)	*p*-value		Median (IQR)/Mean (S.D)	*p*-value
Gender ^a^			Gender^b^		
*Male* *Female*	1.51 (1.27–1.89) 1.50 (1.13–1.98)	0.944	*Male* *Female*	0.75 (0.08) 0.74 (0.14)	0.934
Arterial hypertension ^b^			Arterial hypertension^b^		
*No* *Yes*	1.56 (0.49) 1.66 (0.52)	0.727	*No* *Yes*	0.73 (0.09) 0.76 (0.11)	0.641
Diabetes mellitus^b^			Diabetes mellitus^b^		
*No* *Yes*	1.60 (0.37) 1.70 (0.73)	0.731	*No* *Yes*	0.77 (0.11) 0.73 (0.10)	0.447
Nicotine use ^b^			Nicotine use^b^		
*No* *Yes* *Ex-nicotine use*	1.61 (0.57) 1.82 (0.48) 1.43 (0.13)	0.570	*No* *Yes* *Ex-nicotine use*	0.73 (0.09) 0.83 (0.13) 0.73 (0.04)	0.204
DMARDs ^b^ *No*	1.70 (0.51)	0.338	DMARDs^b^ *No*	0.77 (0.11)	0.340
*Yes*	1.44 (0.50)		*Yes*	0.71 (0.08)	
Statin therapy ^b^			Statin therapy^b^		
*No* *Yes*	1.70 (0.56) 1.48 (0.13)	0.192	*No* *Yes*	0.76 (0.12) 0.73 (0.03)	0.272
CRP positive ^b^			CRP positive^b^		
*No* *Yes*	1.49 (0.38) 1.73 (0.60)	0.317	*No* *Yes*	0.72 (0.07) 0.76 (0.08)	0.255
Cardiovascular events ^b^			Cardiovascular events^b^		
*No* *Yes*	1.61 (0.59) 1.48 (0.10)	0.434	*No* *Yes*	0.74 (0.13) 0.73 (0.03)	0.778

Quantitative characteristics: Spearman’s (a) (non-normal distribution, rho) and Pearson’s (b) (normal distribution, r). Qualitative characteristics: (a) non-normal distribution: presentation as median (interquartile range); (b) normal distribution: presentation as mean (standard deviation)

*cfPWV* carotid-femoral pulse wave velocity, *BMI* Body Mass Index, *HDL* high-density lipoprotein, *LDL* low-density lipoprotein, *TAG* triacylglycerol, *SAP *systolic arterial pressure, *DAP* diastolic arterial pressure, *MAP* mean arterial pressure, *cIMT* carotid intima-media thickness, *CCA *common carotid artery, *ICA* internal carotid artery, *ECA* external carotid artery, *RI* resistance index, *PI* pulsatility index, *DOAC* novel oral anticoagulants, *eGFR* estimated glomerular filtration rate, *ESR* erythrocyte sedimentation rate, *CRP* C-reactive protein

*-*** Significant between the two groups (*p <0.05; ***p <0.001)

**Table 7:** Associations between ECA-PI/ CCA-RI and patients’ characteristics

	ECA-PI			ECA-RI	
	Rho/*r*	*p*-value		Rho/*r*	*p*-value
Age^a^ *(years)*	0.373	0.096	Age^a^ *(years)*	0.420	0.065
BMI^a^ *(kg/m*^*2*^*)*	-0.312	0.180	BMI^a^ *(kg/m*^*2*^*)*	-0.110	0.654
Height^a^ *(cm)*	0.051	0.836	Height^b^ *(cm)*	-0.075	0.768
Weight^a^ *(kg)*	-0.523	0.022	Weight^b^ *(kg)*	-0.347	0.159
HDL^a^ *(mg/dl)*	-0.134	0.647	HDL^a^ *(mg/dl)*	-0.032	0.918
LDL^a^ *(mg/dl)*	0.022	0.940	LDL^a^ (*mg/dl)*	-0.037	0.904
Total cholesterol^a^ *(mg/dl)*	-0.346	0.135	Total cholesterol^b^ *(mg/dl)*	-0.412	0.080
TAG^a^ *(mg/dl)*	-0.171	0.470	TAG^a^ (*mg/dl)*	-0.410	0.081
SAP-DAP ^a^ *(mmHg)*	0.254	0.308	SAP-DAP^b^ *(mmHg)*	0.408	0.104
SAP ^a^ *(mmHg)*	0.283	0.255	SAP^b^ *(mmHg)*	0.447	0.072
DAP^a^ *(mmHg)*	0.158	0.532	DAP^a^ *(mmHg)*	0.189	0.467
MAP^a^ (*mmHg)*	0.235	0.348	MAP^a^ (*mmHg*)	0.376	0.137
cfPWV^a^ *(m/s)*	0.070	0.782	cfPWV^a^ *(m/s)*	0.033	0.901
cIMT ^a^ *(mm)*	-0.075	0.745	cIMT^b^ (*mm)*	-0.122	0.607
Calcification area^ay^	0.008	0.971	Calcification area^a^	0.071	0.767
CCA-PI ^a^	0.327	0.148	CCA-PI^b^	0.418	0.067
CCA-RI ^a^	0.339	0.133	CCA-RI^b^	0.557	0.011*
ICA-PI^a^	0.358	0.111	ICA-PI^a^	0.577	0.008*
ICA-RI ^a^	0.234	0.308	ICA-RI^b^	0.271	0.248
ECA-PI^a^			ECA-PI^a^	0.725	< 0.001***
ECA-RI ^a^	0.725	< 0.001***	ECA-RI^b^		
Prednisolone equivalent^a^ *(mg)*	0.140	0.555	Prednisolone equivalent^a^ (*mg)*	0.319	0.184
eGFR ^a^ *(ml/min)*	0.502	0.024*	eGFR^b^ *(ml/min)*	0.288	0.231
ESR^a^ *(mm/h)*	-0.162	0.521	ESR^a^ (*mm/h)*	-0.404	0.108
Disease duration^a^ (*months)*	-0.074	0.757	Disease duration^a^ *(months)*	-0.040	0.862

	Median (IQR)/Mean (S.D.)	*p*-value		Median (IQR)/Mean (S.D.)	*p*-value
Gender ^a^			Gender^b^		
Male Female	2.94 (2.75–3.21) 2.47 (2.23–3.18)	0.181	Male Female	0.89 (0.04) 0.86 (0.06)	0.215
Arterial hypertension ^a^			Arterial hypertension^b^		
No Yes	2.62 (2.34–3.07) 2.95 (2.46–3.34)	0.458	No Yes	0.88 (0.05) 0.88 (0.05)	0.963
Diabetes mellitus^b^			Diabetes mellitus^b^		
No Yes	2.79 (0.50) 3.25 (1.17)	0.232	No Yes	0.89 (0.04) 0.88 (0.05)	0.761
Nicotine use ^a^			Nicotine use^b^		
No Yes Ex-nicotine use	3.07 (2.52–3.28) 2.62 (2.47–2.95) 2.75 (2.47–2.83)	0.393	No Yes Ex-nicotine use	0.90 (0.04) 0.87 (0.05) 0.86 (0.02)	0.330
DMARDs ^a^			DMARDs^b^		
No Yes	2.90 (2.46–3.34) 2.95 (2.34–3.19)	0.896	No Yes	0.77 (0.11) 0.71 (0.08)	0.535
Statin therapy ^a^			Statin therapy^b^		
No Yes	0.75 (0.69–0.83) 0.73 (0.70–0.75)	0.508	No Yes	0.88 (0.05) 0.90 (0.04)	0.497

Quantitative characteristics: Spearman’s (a) (non-normal distribution, rho) and Pearson’s (b) (normal distribution, r). Qualitative characteristics: (a) non-normal distribution: presentation as median (interquartile range); (b) normal distribution: presentation as mean (standard deviation)

*cfPWV* carotid-femoral pulse wave velocity, *BMI* Body Mass Index, *HDL* high-density lipoprotein, *LDL* low-density lipoprotein, *TAG* triacylglycerol, *SAP *systolic arterial pressure, *DAP* diastolic arterial pressure, *MAP* mean arterial pressure, *cIMT* carotid intima-media thickness, *CCA *common carotid artery, *ICA* internal carotid artery, *ECA* external carotid artery, *RI* resistance index, *PI* pulsatility index, *DMARDs *disease-modifying-anti-rheumatic drugs, *eGFR* estimated glomerular filtration rate, *ESR* erythrocyte sedimentation rate, *CRP* C-reactive protein

*-*** Significant between the two groups (*p <0.05; ***p <0.001)

### Association between group status (patients vs. controls): cfPWV, Doppler indices, cIMT, and plaques

In unadjusted analyses, cfPWV was significantly higher in PMR patients compared with controls [9.21 (1.81) vs. 7.19 (1.38); *p* < 0.001]. This difference remained significant after adjustment in a linear regression model for sex, diabetes mellitus, age, arterial hypertension, and smoking (β = 0.581; 95% CI: 0.114 to 1.047; p_adj_ = 0.015).

**Fig. 1:**
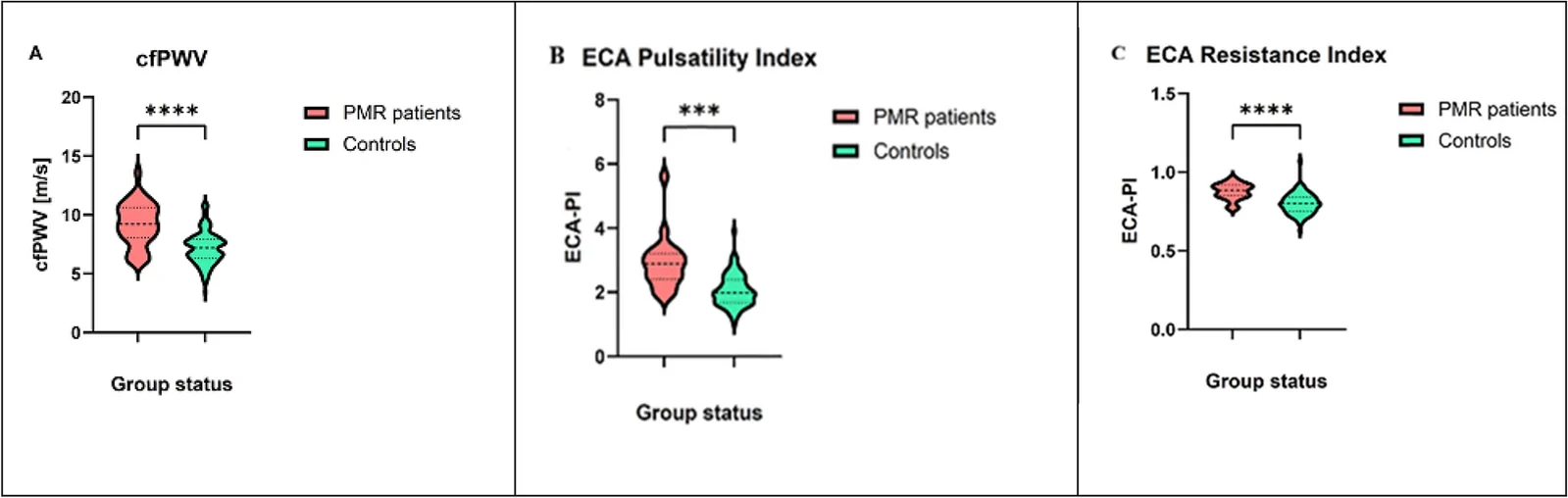
Comparison of cfPWV, ECA-PI and ECA-RI between polymyalgia rheumatica patients and controls. cfPWV: carotid-femoral pulse wave velocity, PI: pulsatility index, RI: resistance index, ECA: external carotid artery, (red: polymyalgia rheumatica patients; blue: controls)

In unadjusted analyses, CDUS-derived markers pointed to significant differences between controls and patients for CCA-RI [0.77 (0.74–0.82) vs. 0.74 (0.69–0.77); *p* = 0.008], ICA-PI [1.50 (1.21–1.88) vs. 1.22 (0.96–1.54); *p* = 0.004], ICA-RI [0.74 (0.11) vs. 0.66 (0.09); *p* < 0.001], ECA-PI [2.90 (2.41–3.20) vs. 1.99 (1.69–2.39); *p* < 0.001] and ECA-RI [0.89 (0.85–0.92) vs. 0.80 (0.75–0.84); *p* < 0.001]. Similarly, cIMT [0.99 (0.82–1.20) vs. 0.78 (0.67–0.91); *p* < 0.001], plaque area [0.12 (0.02–0.41) vs. 0.00 (0.00-0.06); *p* < 0.001] and SCA [84.6% [22] vs. 44.8% [36]; *p* < 0.001] differed significantly between groups, in unadjusted analyses (Fig. 1).

After adjustment for the aforementioned CV risk factors, several vascular parameters remained significantly altered in PMR patients: plaque area (β = 0.469; 95% CI: 0.027–0.246; p_adj_ = 0.007), ICA-RI (β = 0.077; 95% CI: 0.017–0.138; p_adj_ = 0.012), ECA-PI (β = 0.170; 95% CI: 0.131–0.807; p_adj_ = 0.007), and ECA-RI (β = 0.056; 95% CI: 0.017–0.095; p_adj_ = 0.005). However, cIMT (β = 0.059; 95% CI: -0.027–0.145; p_adj_ = 0.176), CCA-PI (β = 0.080; 95% CI: -0.143–0.304; p_adj_ = 0.477), CCA-RI (β = 0.023; 95% CI: -0.020–0.067; p_adj_ = 0.282) and ICA-PI (β = 0.227; 95% CI: -0.057–0.510; p_adj_ = 0.116) were no longer significantly different after adjustment.

### Propensity Score Matching (PSM)

PSM was performed to balance differences in age, sex, smoking status, arterial hypertension, and diabetes mellitus (full model in supplementary material S1 and S2). In the overall cohort, 71 matched pairs were identified using genetic matching with replacement, whereas 16 matched pairs were obtained in the sonography subgroup without replacement.

Consistent with the linear regression model, statistically significant differences were observed for cfPWV [9.20 (8.06–10.64) vs. 8.20 (7.70–9.28); p_adj_ = 0.012], ECA-PI [2.62 (0.50) vs. 2.15 (0.54); p_adj_ = 0.027], and ECA-RI [0.87 (0.05) vs. 0.81 (0.07); p_adj_ = 0.020]. In contrast to the linear regression model, cumulative plaque area [0.08 (0.00-0.17) vs. 0.00 (0.00-0.15); p_adj_ = 0.120] and ICA-RI [0.75 (0.13) vs. 0.69 (0.08); p_adj_ = 0.169] were no longer statistically significantly different after PSM. Furthermore, PSM demonstrated a greater quantitative plaque burden [12 (75.0%) vs. 6 (37.5%); p_adj_ = 0.033] and a higher prevalence of SCA [13 (81.3%) vs. 7 (43.8%); p_adj_ = 0.028] in patients compared with controls.

### Associations of cfPWV and carotid sonography markers with patient group and disease characteristics

Among PMR patients, cfPWV was associated with age (*r* = 0.588, *p* < 0.001), mean arterial pressure (MAP) (rho = 0.247, *p* = 0.038) and systolic blood pressure (SAP) (rho = 0.331, *p* = 0.005). CIMT correlated with age (*r* = 0.542, *p* = 0.004), cfPWV (*r* = 0.519, *p* = 0.019), MAP (*r* = 0.515, *p* = 0.020) and weight (rho = 0.432, *p* = 0.044). Patients treated with DMARDs had lower values for cIMT [0.77 (0.54–0.96) vs. 0.99 (0.93–1.20), *p* = 0.024], whereas higher cIMT values were observed in statin-treated patients [1.25 (0.27) vs. 0.91 (0.22), *p* = 0.003] and patients with prior CVD events [1.22 (0.34) vs. 0.93 (0.21), *p* = 0.020]. However, the association of cIMT with DMARD therapy turned insignificant when controlled for the effects of age (p_adj_=0.203).

Cumulative plaque area was linked to age (*r* = 0.662, *p* < 0.001), cfPWV (*r* = 0.812, *p* < 0.001), SAP (*r* = 0.686, *p* < 0.001), and MAP (*r* = 0.596, *p* = 0.006) and was also significantly higher in patients with arterial hypertension [0.38 (0.09–0.59) vs. 0.00 (0.00–0.05), *p* = 0.015] and in those with elevated CRP levels [0.41 (0.13–1.07) vs. 0.05 (0.00–0.31), *p* = 0.016]. Moreover, CCA-RI correlated with age (*r* = 0.593, *p* = 0.005).

## Discussion

In this exploratory cross-sectional analysis, PMR was associated with a pattern of macrovascular dysfunction characterised by increased aortic stiffness and elevated ECA pulsatility and resistance relative to controls. Importantly, both systemic inflammation (as reflected by CRP) and patient-related factors (age and MAP) correlated with these measures, suggesting that inflammatory burden and haemodynamic load may contribute to the observed vascular alterations.

To our knowledge, this is the first study to simultaneously evaluate a broad panel of oscillometric and sonographic markers of macrovascular damage in PMR, and one of the largest investigations of vascular surrogate outcomes in this entity. In the general population, elevated pulse wave velocity (PWV) is consistently associated with incident CV events, CV morbidity, and all-cause mortality [22–24]. Against this background, our findings support the concept that PMR may be linked to an unfavourable vascular phenotype and, potentially, increased CV risk. Beyond its prognostic relevance, aortic stiffness integrates cumulative structural and functional arterial wall changes, including medial fibrosis, elastin fragmentation, endothelial dysfunction, and inflammation-driven remodelling [30].

In line with this interpretation, previous studies have also reported increased arterial stiffness in PMR using different methodological approaches. The cardio-ankle vascular index (CAVI), a stiffness parameter considered less dependent on blood pressure [31], was elevated in a cohort of 48 PMR patients [32]. Moreover, two studies found higher PWV in 39 and 65 PMR patients compared with matched controls [33, 34], and both reported a reduction in PWV during follow-up under glucocorticoid therapy, suggesting at least partial reversibility of arterial stiffness in PMR. This improvement may reflect decreasing systemic inflammatory activity, potentially modulated by direct vascular effects of glucocorticoids. Notably, one of these studies included both PMR and giant cell arteritis (GCA) patients [34]. In contrast, we deliberately excluded PMR patients with concomitant GCA to specifically assess the vascular alterations in patients with clinically isolated PMR. Our data therefore suggest that macrovascular dysfunction in PMR can be present even in the absence of clinically overt large-vessel vasculitis. Nevertheless, PMR and GCA share overlapping inflammatory and vascular mechanisms, and complete pathophysiological separation of both conditions may not always be possible. Therefore, a contribution of subclinical vasculitic processes to the observed vascular alterations cannot be entirely excluded.

In our study, carotid intima–media thickness (cIMT) did not differ between groups after adjustment for confounders. This finding contrasts with a previous report [32]. Most prior investigations have assessed cIMT primarily in the context of vasculitic involvement [35], suggesting that increased cIMT may, at least in part, reflect inflammation-related vascular wall changes rather than true atherosclerotic burden. Moreover, accumulating evidence has raised concerns regarding the validity of cIMT as a surrogate marker of atherosclerosis. High-risk plaques most commonly develop in the carotid bulb or internal carotid artery (ICA), rather than at the common carotid artery (CCA) measurement site typically used for cIMT assessment [25]. In addition, intima thickening may reflect vascular remodelling related to ageing and hypertension rather than atherosclerosis per se [36].

In contrast, plaque burden has been shown to provide stronger predictive value for future CV events than cIMT alone [25], and plaque area is considered an independent predictor of future coronary heart disease events [37]. In our analysis, cumulative plaque area differed significantly between groups in the linear regression model but did not retain statistical significance after PSM. Nevertheless, plaque area values remained numerically higher in the PMR group following PSM. Furthermore, PSM analysis revealed a higher prevalence of plaque presence and SCA in patients with PMR. Overall, our findings suggest an increased burden of atherosclerotic changes in PMR.

Adjusted statistical analyses using linear regression and PSM demonstrated higher ECA-PI and ECA-RI values in patients with PMR compared with healthy controls. In addition, ICA-RI was significantly increased in the linear regression analysis, whereas CDUS parameters of the CCA as well as ICA-PI, did not differ between groups. The role of compliance indices derived from the ECA has been less extensively studied than those of the CCA or ICA, largely because the ECA seems to be less commonly implicated in cerebrovascular events. However, the ECA supplies high-resistance vascular beds of the facial and scalp tissues [38], and through collateral pathways can contribute to cerebral blood circulation, particularly in the setting of severe carotid stenosis [39] and acute stroke [40]. Notably, Bai et al. [41] reported increased RI values across all three carotid arteries in patients with acute and chronic ischemic stroke, even after adjustment for CV risk factors and carotid plaque burden, suggesting that these Doppler parameters may provide additional information on vascular function. The ICA, as the primary supplier of cerebral blood flow, is the most clinically relevant vessel for stroke risk assessment. Increased ICA-RI has been identified as an independent predictor of CV events, including acute myocardial infarction, stroke, and cardiovascular death [42]. Staub et al. [42] further demonstrated that the predictive value of ICA-RI for CV morbidity and mortality is at least comparable to that of cIMT. However, since PI and RI are not well-examined prognostic parameters, they should be interpreted in conjunction with better validated markers of vascular damage. Therefore, we did not rely solely on the indices but combined them with established vascular imaging and functional biomarkers. The rationale for this multimodal vascular assessment was to capture complementary aspects of vascular health, since vascular damage may affect different arterial beds heterogeneously. Importantly, the inclusion of PI and RI represents a novel aspect of our study, as these parameters have not been investigated in this context. By integrating these emerging biomarkers with established vascular measures, our study provides new insights into their potential complementary value for the assessment of vascular damage.

Beyond group differences, the observed vascular surrogate markers also showed clinically plausible associations with patient- and disease-related factors. Consistent with our results, Emamifar et al. [34] reported that cfPWV correlated with MAP, SAP, and age. In the general population, age and blood pressure are well established as the two principal independent determinants of cfPWV [43]. Age-related increases in aortic stiffness are largely attributed to progressive loss of aortic elasticity during vascular ageing, driven by elastin degradation and structural arterial wall remodelling [43, 44].

The relationship between cfPWV and blood pressure is complex and likely bidirectional: increased arterial stiffness may contribute to higher systolic pressure, while hypertension itself accelerates arterial stiffening. Earlier pulse wave reflection in stiffened arteries, with systolic wave augmentation, has been proposed as one mechanism linking stiffness to systolic blood pressure elevation. Similar associations with age and hypertension have been described for GSUS-derived markers [36, 45].

We further observed a higher cumulative plaque area in PMR patients with elevated CRP levels compared with those below the predefined cut-off, supporting the concept that systemic inflammation may be associated with atherosclerotic burden. This finding is biologically plausible, as chronic low-grade inflammation can promote atherogenesis by driving endothelial dysfunction, increasing oxidative stress, and facilitating monocyte recruitment and foam-cell formation. In parallel, pro-inflammatory cytokines can destabilise vascular homeostasis and accelerate smooth muscle cell activation, extracellular matrix remodelling, and arterial wall thickening, processes that contribute to plaque progression and expansion [46].

Interestingly, patients receiving DMARD therapy prior to study inclusion showed lower cIMT values, which may suggest a protective effect of sustained anti-inflammatory treatment on vascular wall changes. A similar association between DMARD use and reduced cIMT has been reported in rheumatoid arthritis [47]. However, this finding should be interpreted cautiously, as DMARD-treated patients were significantly older; therefore, causality cannot be inferred, and longitudinal studies are needed to clarify this relationship. In contrast, higher cIMT values were observed in statin-treated patients, which likely reflects confounding by indication, with statin therapy serving as a marker of pre-existing dyslipidaemia and higher baseline atherosclerotic risk rather than a treatment-related adverse effect.

Our exploration has some limitations. First, we did not assess the prognostic impact of CV surrogate markers on future mortality or morbidity. However, both cfPWV and carotid sonography have previously demonstrated strong predictive value for CV events [22]. Second, PMR patients exhibited a higher prevalence of traditional CV risk factors than controls, which may have introduced bias. We addressed this imbalance using propensity score matching and multivariable regression, and we report adjusted results; however, residual confounding cannot be excluded. Furthermore, the relatively low number of matched pairs in the ultrasound subgroup (*n* = 16 ) may limit statistical power. Therefore we additionally used linear regression analyses to support the robustness of the analysis. Nevertheless, the findings should be interpreted as exploratory and hypothesis-generating rather than conclusive, and should be validated in larger studies. Third, glucocorticoid therapy may have influenced vascular measures. Although we observed no significant association between current glucocorticoid use and surrogate markers, the effects of cumulative exposure cannot be ruled out; longitudinal studies incorporating cumulative dose are warranted. Fourth, GCA was excluded primarily on clinical grounds, which may not fully preclude subclinical vasculitis, given the overlap in pathophysiological mechanisms between PMR and GCA. Fifth, the ultrasound subgroup was relatively small because carotid imaging was implemented later within the CARD cohorts; nevertheless, recruitment was consecutive, and the sample size provided sufficient power for the performed analyses.

In conclusion, this study provides the first integrated evaluation of a broad panel of surrogate markers of angiopathy and atherosclerosis in patients with PMR. To our knowledge, it is also the first to combine vascular stiffness assessment, carotid Doppler indices, and plaque burden measurements in this population. We found increased aortic stiffness together with elevated ECA pulsatility and resistance compared with controls. Given their non-invasive, radiation-free, and readily applicable nature, these vascular markers may support CV risk stratification in PMR. However, larger prospective longitudinal studies are needed to confirm these findings and to determine their prognostic value for future CV outcomes.

## Supplementary Information

Supplementary Material 1.

## Data Availability

The datasets used and analysed during the current study are available from the corresponding author on reasonable request.

## Abbreviations

PMR: Polymyalgia rheumatica
CfPWV: Carotid-femoral pulse wave velocity
GSUS: Greyscale Doppler ultrasound
CDUS: Colour Doppler ultrasound
Cimt: Carotid intima-media thickness
RI: Resistance index
PI: Pulsality index
CCA: Common carotid artery
ICA: Internal carotid artery
ECA: External carotid artery
MLR: Multivariable liner regression
PSM: Propensity score matching
CVD: Cardiovascular disease
CV: Cardiovascular
GCA: Giant cell arteritis
SCORE: Systematic Coronary Risk Evalutaion
PROCAM: Prospective Cardiovascular Münster
POLYMYACARD: POLYMYalgia Associated CARDiovascular risk
BMI: Body mass index
DMARDs: Disease-modifying antirheumatic drugs
NSAIDs: Non-steroidal anti-inflammatory drugs
DOACs: Direct oral anticoagulants
CRP: C-reactive protein
ESR: Erythrocyte sedimentation rate
eGFR: Estimated glomerular filtration rate
MAP: Mean arterial pressure
SAP: Systolic blood pressure
