# Supplementary material for: Macrovascular damage in polymyalgia rheumatica: integrated vascular phenotyping in the POLYMYACARD cohort
Source: Arthritis Res Ther. 2026 Sep 15;28:170. doi: 10.1186/s13075-026-03886-8 (PMC13576255; doi:10.1186/s13075-026-03886-8)
Supplement: Supplementary file 1 [file 13075_2026_3886_MOESM1_ESM.docx]

**S 1:** Propensity score matching for total group (genetic matching with replacement)

| **All subjects** | **Patients (n=71)** | **Controls (n=71)** | **p value** | **Standardized Mean Difference** |
| --- | --- | --- | --- | --- |
| **Age**^a^ *(years)* | 69.00 (60.00-74.00) | 64.00 (59.00-75.00) | 0.200 | 0.20 |
| **Gender** *(female)* | 43/71 (60.6%) | 36/71 (50.7%) | 0.237 | 0.20 |
| **Arterial hypertension** *(yes)* | 45/71 (63.4%) | 41/71 (57.7%) | 0.492 | 0.12 |
| **Diabetes mellitus** *(yes)* | 19/71 (26.8%) | 10/71 (14.1%) | 0.061 | -0.32 |
| **Nicotine use** *(smoker)*  *(ex-smoker)* | 8/71 (11.3%)  9/71 (12.7%) | 8/71 (11.3%)  9/71 (12.7%) | 1.000 | 0.00  0.00 |
| **cfPWV**^a^ (MW) | 9.20 (8.06-10.64) | 8.20 (7.70-9.28) | 0.012* |  |

^a^Non-normal distribution: presentation as median (interquartile range). ^b^Normal distribution: presentation as mean (S.D.). cfPWV carotid-femoral pulse wave velocity

**S 2:** Propensity Score Matching for sonography subgroup (genetic matching without replacement)

| **All subjects** | **Patients (n=16)** | **Controls (n=16)** | **p value** | **Standardized Mean Difference** |
| --- | --- | --- | --- | --- |
| **Age**^b^ *(years)* | 62.38 (9.83) | 62.94 (10.38) | 0.876 | -0.06 |
| **Female** *(yes)* | 10/16 (62.5%) | 10/16 (62.5%) | 1.000 | 0.00 |
| **Arterial hypertension** *(yes)* | 7/16 (43.8%) | 7/16 (43.8%) | 1.000 | 0.00 |
| **Diabetes mellitus** *(yes)* | 2/16 (12.5%) | 1/16 (6.3%) | 0.544 | 0.21 |
| **Nicotine use** *(smoker)*  *(ex-smoker)* | 5/16 (31.3%)  0/16 (0.0%) | 6/16 (37.5%)  0/16 (0.0%) | 0.710 | -0.13  0.00 |
| **cIMT**^b^ *(mm)* | 0.93 (0.24) | 0.82 (0.12) | 0.141 |  |
| **Plaques** *(yes)* | 12/16 (75.0%) | 6/16 (37.5%) | 0.033* |  |
| **Plaque count**^a^ *(median)* | 1.00 (0.25-2.00) | 0.00 (0.00-1.00) | 0.056 |  |
| **Calcification area**^a^ *(cm^2^)* | 0.08 (0.00-0.17) | 0.00 (0.00-0.15) | 0.120 |  |
| **SCA** *(yes)* | 13/16 (81.3%) | 7/16 (43.8%) | 0.028* |  |
| **CCA-PI**^a^ | 1.77 (1.33-1.99) | 1.60 (1.51-1.76) | 0.263 |  |
| **CCA-RI**^a^ | 0.76 (0.72-0.80) | 0.76 (0.71-0.77) | 0.643 |  |
| **ICA-PI**^a^ | 1.61 (1.17-2.03) | 1.29 (1.13-1.50) | 0.264 |  |
| **ICA-RI**^b^ | 0.75 (0.13) | 0.69 (0.08) | 0.169 |  |
| **ECA-PI**^b^ | 2.62 (0.50) | 2.15 (0.54) | 0.027* |  |
| **ECA-RI**^b^ | 0.87 (0.05) | 0.81 (0.07) | 0.020* |  |

^a^Non-normal distribution: presentation as median (interquartile range). ^b^Normal distribution: presentation as mean (S.D.). cIMT: carotid intima-media thickness; SCA:subclinical carotid atherosclerosis; CCA:common carotid artery; ICA: internal carotid artery; ECA: external carotid artery; RI: resistance index; PI: pulsatility index

*-*** Significant between the two groups.
